# Supplementary material for: Predicting visual function by interpreting a neuronal wiring diagram
Source: Nature. 2024 Oct 2;634(8032):113–23. doi: 10.1038/s41586-024-07953-5 (PMC11446822; doi:10.1038/s41586-024-07953-5)
Supplement: Supplementary file 6 — CRF and ERF predictions for individual TmY4 and TmY9 cells. Analogous to Supplementary Data 3, but for TmY target types. Shown are the top four monosynaptic pathways, the strongest pathway passing through each of the top ten intermediary types (ranking from Extended Data Fig. 7), and the trisynaptic pathway Tm1–TmY–Dm3–TmY (see the section entitled Prediction of spatial normalization). [file 41586_2024_7953_MOESM6_ESM.zip › DataS4/TmY9q/720575940611142257.pdf]

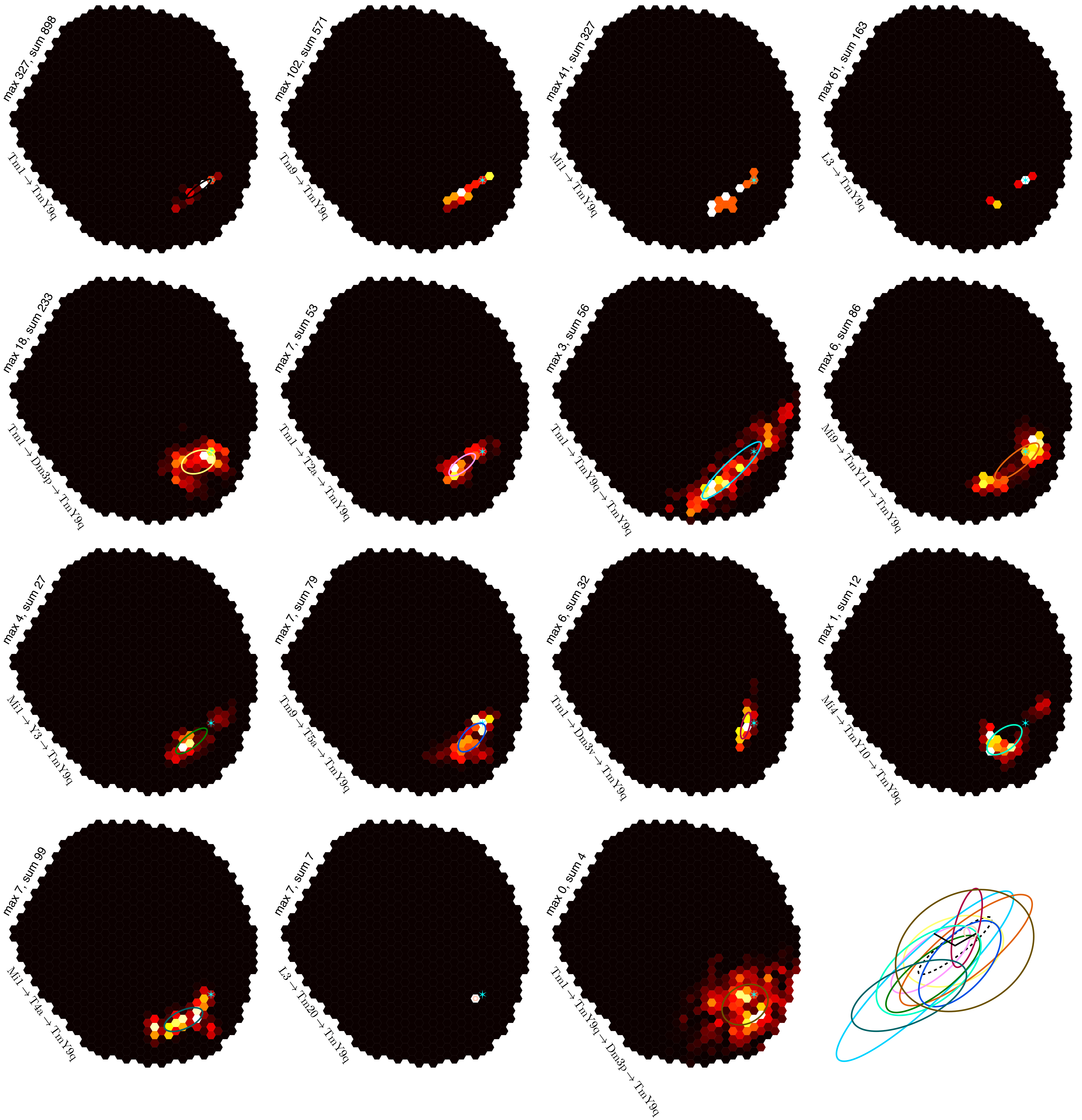
max 327, sum 898
Tm1 → TmY9q
max 102, sum 571
Tm9 → TmY9q
max 41, sum 327
Mi1 → TmY9q
max 61, sum 163
L3 → TmY9q
max 18, sum 233
Tm1 → Dm3p → TmY9q
max 7, sum 53
Tm1 → T2a → TmY9q
max 3, sum 56
Tm1 → TmY9q → TmY9q
max 6, sum 86
Mi9 → TmY11 → TmY9q
max 4, sum 27
Mi1 → Y3 → TmY9q
max 7, sum 79
Tm9 → T5a → TmY9q
max 6, sum 32
Tm1 → Dm3v → TmY9q
max 1, sum 12
Mi4 → TmY10 → TmY9q
max 7, sum 99
Mi1 → T4a → TmY9q
max 7, sum 7
L3 → Tm20 → TmY9q
max 0, sum 4
Tm1 → TmY9q → Dm3p → TmY9q
